# The Use, Standardization, and Interpretation of Brain Imaging Data in Clinical Trials of Neurodegenerative Disorders

**DOI:** 10.1007/s13311-021-01027-4

**Published:** 2021-04-12

**Authors:** Adam J. Schwarz

**Affiliations:** Takeda Pharmaceuticals Ltd., 40 Landsdowne Street, Cambridge, MA 02139 USA

**Keywords:** Imaging, Clinical trials, MRI, PET, Neurology

## Abstract

Imaging biomarkers play a wide-ranging role in clinical trials for neurological disorders. This includes selecting the appropriate trial participants, establishing target engagement and mechanism-related pharmacodynamic effect, monitoring safety, and providing evidence of disease modification. In the early stages of clinical drug development, evidence of target engagement and/or downstream pharmacodynamic effect—especially with a clear relationship to dose—can provide confidence that the therapeutic candidate should be advanced to larger and more expensive trials, and can inform the selection of the dose(s) to be further tested, i.e., to “de-risk” the drug development program. In these later-phase trials, evidence that the therapeutic candidate is altering disease-related biomarkers can provide important evidence that the clinical benefit of the compound (if observed) is grounded in meaningful biological changes. The interpretation of disease-related imaging markers, and comparability across different trials and imaging tools, is greatly improved when standardized outcome measures are defined. This standardization should not impinge on scientific advances in the imaging tools per se but provides a common language in which the results generated by these tools are expressed. PET markers of pathological protein aggregates and structural imaging of brain atrophy are common disease-related elements across many neurological disorders. However, PET tracers for pathologies beyond amyloid β and tau are needed, and the interpretability of structural imaging can be enhanced by some simple considerations to guard against the possible confound of pseudo-atrophy. Learnings from much-studied conditions such as Alzheimer’s disease and multiple sclerosis will be beneficial as the field embraces rarer diseases.

**Supplementary Information:**

The online version contains supplementary material available at 10.1007/s13311-021-01027-4.

## Introduction

Imaging methods are increasingly used in clinical trials of neurological disorders. This has been most evident in Alzheimer’s disease and multiple sclerosis, in which imaging has played an important role in many phase 1, 2, and 3 trials over the past decade or more. The range of neurological conditions studied in interventional trials is likely to increase as drug development programs targeting rarer and genetic disorders increases. From an imaging perspective, two themes apply to many of these diseases and many therapeutic approaches—the use of positron emission tomography (PET) radiotracers to image protein aggregates that are often the neuropathological hallmarks of the disease, and the use of magnetic resonance imaging (MRI) to measure brain atrophy.

Alzheimer’s disease is defined by the presence of amyloid β plaques and neurofibrillary tangles of misfolded tau protein; Huntington’s disease is accompanied by the presence of intranuclear aggregates of mutant huntingtin; synucleinopathies such as Parkinson’s disease, dementia with Lewy bodies, and multiple system atrophy are defined by alpha-synuclein-containing Lewy bodies; ataxias are associated with CAG-repeat protein aggregates; amyotrophic lateral sclerosis is associated with aggregates of transactive response DNA-binding protein 43 kDa (TDP-43); and frontotemporal dementia is associated with aggregates of either 4-repeat (4R) tau, 3-repeat (3R) tau, or TDP-43. The development of PET tracers for amyloid β and, more recently, tau tangles in the past 15 years has revolutionized clinical trials in Alzheimer’s disease—permitting, after a fashion, neuropathology to be performed longitudinally in living persons. These imaging tools are relevant not only because these pathologies are definitive for the disease under study but also because they are often the target of treatment. Efforts are underway to develop PET tracers for other protein aggregates mentioned above, although these are challenged by the generally lower density of the pathological deposits (hence, binding sites)—requiring high-affinity tracers—and the possibility of co-occurrence of co-pathologies (such as amyloid or tau) that may occur, e.g., due to age—requiring high tracer selectivity. Nevertheless, the advent of PET tracers for these other pathologies will be equally enabling for clinical trials in these other disorders.

Most neurological disorders are also associated with a profound loss of brain parenchymal tissue, above and beyond that observed in healthy age-matched controls. The anatomical patterns of this accelerated brain atrophy are disease-specific and relate to the particular symptoms that are characteristic of each disease. These changes in regional brain volume are readily detectable in living individuals using volumetric MRI (vMRI) scans. vMRI measures of brain atrophy are generally very well-behaved in a natural history context, related to disease severity, and correlated with clinical scales, symptomatic decline, and neuropathology (e.g., neuron density). They have robust longitudinal measurement characteristics and are generally well-powered as biomarkers to detect treatment effects. Suitable MRI scanners are widely available, and vMRI scans have been successfully incorporated in many large, global, pharma-sponsored phase 2 and 3 trials. It is reasonable to expect that any successful disease-modifying therapy would slow the rate of brain atrophy, providing evidence that the disease process has been slowed. In multiple sclerosis, brain atrophy is increasingly studied in addition to the more traditional quantification of the number and volume of T2 lesions.

Similar to atrophy, most neurological disorders are associated with a specific pattern of glucose hypometabolism detectable using [^18^F]fluorodeoxyglucose (FDG)-PET. Imaging biomarkers based on FDG-PET generally have good operating characteristics but are often de-prioritized in favor of more molecularly-specific PET tracers, due to the need for additional radioactive exposure and subject visit and the fact that, as a measure of brain function, FDG-PET can also be influenced by environmental factors including symptomatic therapies. Neuroinflammation is increasingly being identified as a relevant aspect of disease biology in many neurological disorders, primarily using TSPO PET ligands to date. However, much remains to be understood about its role in disease biology. Emerging evidence of a potential role of synaptic density or plasticity could provide another tool, but at the present time, the evidentiary basis is still accumulating.

In this review, we discuss the use, standardization, and interpretation of imaging in clinical trials. We concentrate particularly on PET imaging of aggregated proteins and MR imaging of brain atrophy. Although many of the considerations may generalize, the scope is trials of novel pharmacological treatments for neurology in drug development (phases 1–3). We also briefly discuss the various roles of imaging in a broader context of fluid biomarkers, including cost and practicality considerations and the trade-offs between them.

## Use of Imaging in Clinical Trials

**Fig. 1:**
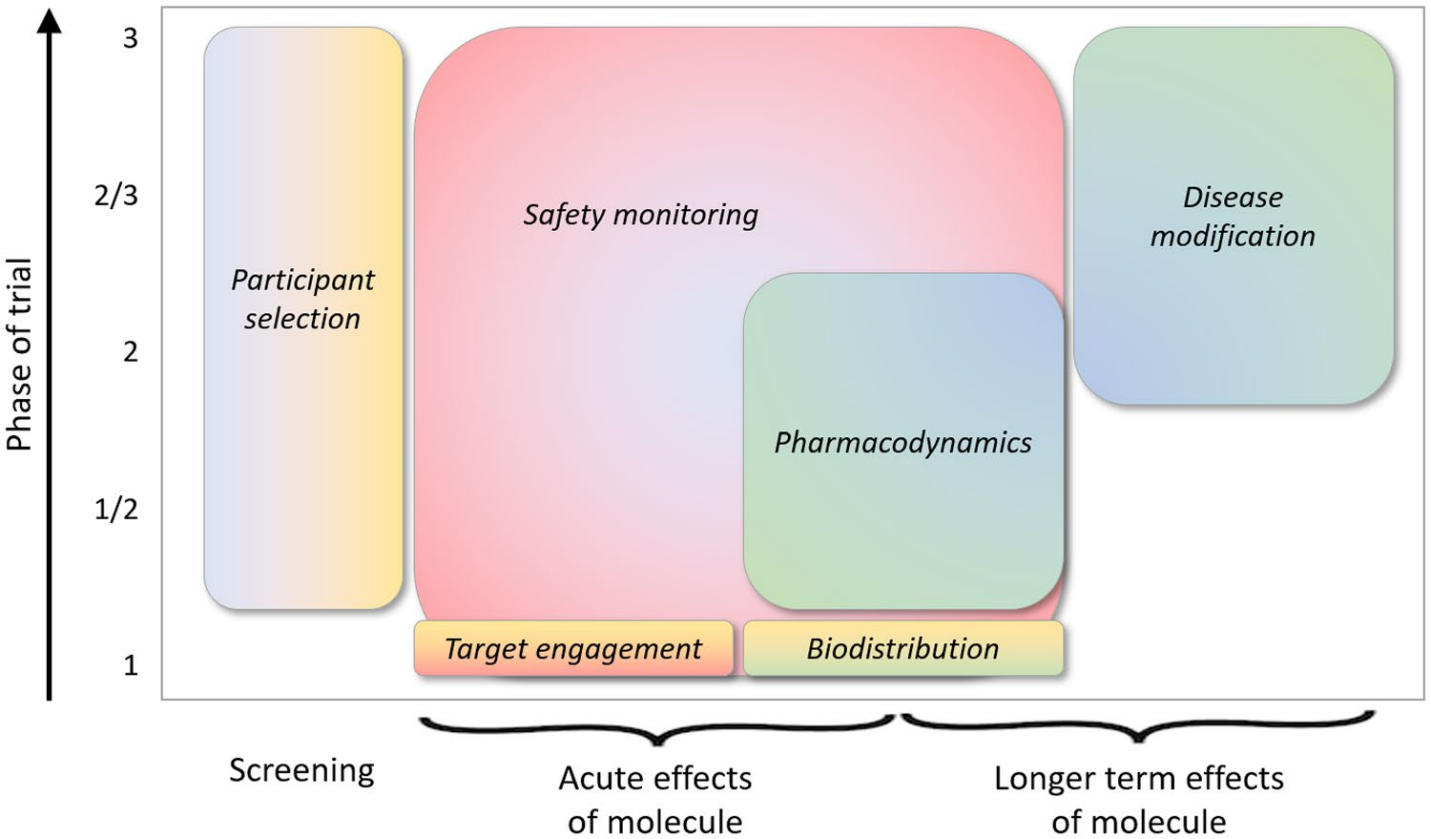
Schematic overview of applications of imaging in clinical trials for drug development. (This is intended to indicate typical scenarios, but exceptions may occur.)

Imaging has a number of uses in clinical trials for drug development (Fig. 1). These comprise application in the screening stage, as an inclusionary or exclusionary criterion, and its use as an outcome measure, to provide information on the interaction between the candidate therapeutic molecule and the biological system it is targeting. These categories are consistent with the BEST (Biomarkers, EndpointS, and other Tools) glossary developed by the US Food and Drug Administration (FDA) and National Institutes of Health (NIH) [1] in the context of biomarker qualification, in which a biomarker is approved by regulators for a specific drug development context-of-use. Regulatory qualification is not a prerequisite for the use of an imaging biomarker in a clinical trial, but it does signal a degree of evidentiary maturity and confidence in its interpretation.

### Imaging as a screening Tool: Patient Selection, Enrichment, and Personalized Medicine

#### Confirmation of Disease Biology

##### Sporadic Diseases

Imaging biomarkers are being increasingly used to refine the selection of patients (or unaffected but at-risk participants) for clinical trials. In the case of sporadic diseases (i.e., with no known fully penetrant genetic cause), while clinical diagnostic guidelines remain the foundation of enrollment criteria, they do not provide full biological specificity. The advent of PET tracers specific for the insoluble protein aggregates that provide the definitive post mortem biological diagnosis of many neurological disorders has revolutionized clinical trials in this area of drug development.

Alzheimer’s disease (AD) provides a good illustration of this. AD is defined neuropathologically by the presence of amyloid plaques and tau neurofibrillary tangles. However, the prevalence of amyloid positivity by PET in clinically diagnosed AD is age- and APOE ɛ4-dependent in the range of 80–90% [2, 3]. Consistent with this, among the first phase 3 AD trials that included amyloid PET sub-studies but did not use amyloid positivity as an inclusion criterion, 15–22% of participants clinically diagnosed with mild-moderate AD were amyloid negative on PET [4, 5]. Other biological insults are thus driving cognitive decline and manifesting as a phenocopy of Alzheimer’s disorder in some individuals [6, 7]. In comparison, between about 15 and 50% of older individuals without cognitive impairment may be amyloid positive, and hence at the early stages of the AD pathological cascade, whereas individuals with mild cognitive impairment have an intermediate prevalence of amyloid positivity [3, 8].

Amyloid PET tracers were thus quickly adopted in AD drug development to confirm the presence of amyloid β in the screening phase of clinical trials, to ensure that only amyloid-positive subjects were enrolled [9–11]. Not only does this ensure the presence of one of the defining pathologies of AD, but, in the case of anti-amyloid treatments, it ensures that the target pathology of the therapeutic intervention is present in the brains of the study participants. This maximizes the chance of a therapeutic benefit and avoids needless risk of exposure to the compound by participants who cannot derive any benefit from it. Moreover, amyloid PET has become an enabling technology for studies in earlier phases of the pathological cascade, before the onset of clinical symptoms [12, 13].

More recently, the advent of tau PET tracers has enabled a similar approach to be performed for trials with tau-directed therapies in AD. Whereas the appearance of amyloid plaques is a very early event in the Alzheimer’s pathological cascade [14], tau tangles correlate—both anatomically and temporally—much more closely with clinical symptoms and evidence a more heterogeneous distribution within the brain. The ability to measure both amyloid and tau deposits in vivo has enabled the development of research frameworks closely tied to the biological definition of the disease [15, 16]. Beyond AD, tau PET has potential utility for participant selection or staging in other primary tauopathies, especially those for which the molecular driver is not well-defined clinically and/or genetically. For example, most cases of sporadic FTD are due to either TDP-43 or tau pathology, and so the availability of a tau PET tracer able to detect 4R or 3R tau deposits would be an enabling technology for trials targeting either pathology—patients could be screened in or out based on a tau PET scan, depending on the target. The first tau PET tracers were optimized to bind to “AD tau,” which comprises a mix of 3R and 4R proteins in a paired-helical filament conformation. However, other tauopathies, including animal models, can present with tau deposits of a different protein composition and/or conformation. For example, tau deposits found in FTD are typically comprised of pure 4R or 3R tau, to which some but not all current tau tracers appear to bind strongly [17, 18]. PET tracers are also being developed to image insoluble aggregates characteristic of other proteinopathies—for example, mutant huntingtin, TDP-43, and alpha-synuclein—and, when available, are likely to be equally impactful for drug development.

Visual read paradigms to assign a scan as positive or negative have been developed for amyloid and more recently tau PET tracers and validated against neuropathology in autopsy studies [19–21]. While visual reads are sometimes used as the inclusion methodology in clinical trials, quantitative PET scan analysis criteria are also often used, either in conjunction with or instead of visual reads [22]. Most simply, a PET scan is summarized by a single global or representative derived number, which as a continuous variable can then be binarized with respect to a positivity threshold value. These cutoff values can be determined by maximizing agreement with visual reads [23, 24] or autopsy data [23], or with respect to deviation beyond the normal range in an appropriate control cohort [25]. The thresholds and brain regions used may be influenced by other clinical and genetic factors [26] and should be carefully considered based on the disease stage being targeted [27] (see also the “Staging” section).

At the present time, three amyloid tracers ([^18^F]florbetapir, [^18^F]florbetaben, and [^18^F]flutemetamol) have received regulatory approval for the purposes of estimating amyloid β neuritic plaque density in adult patients being evaluated for AD and other causes of cognitive decline, and one tau tracer ([^18^F]flortaucipir) is FDA-approved for the purposes of estimating the density and distribution of aggregated tau NFTs in patients with cognitive impairments who are being evaluated for AD. Such approval is not a precondition for the use of these (or other) imaging tools in drug development, but it is required for the use of such tracers in clinical practice, e.g., as companion diagnostics.

##### Genetic Diseases

In the case of the genetically determined neurological diseases (such as Huntington’s disease, spinocerebellar ataxias, or the autosomal dominant variants of Alzheimer’s disease and frontotemporal dementia), a PET scan or fluid biomarker assay to assure the correct biological diagnosis is not usually necessary. The clinical diagnosis can be confirmed instead by the relevant genetic test, and the presence of the target pathology can be safely assumed. However, there may still be utility in assessing the load or stage of the pathological burden, as this may be a factor in the response to treatment. Moreover, when used as an outcome biomarker, a baseline PET scan will still be necessary.

#### Staging

A more nuanced use of these molecular imaging tools is that of staging the severity of the disease, based on the extent or load of pathology in different brain regions. In this approach, a more granular categorization of a PET scan is derived, beyond just classifying it as “positive” or “negative” for the pathology of interest.

In AD, Aβ PET scans are generally well-characterized by a global measure of cortical amyloid load, typically indexed by sampling regions of the cortex [19, 28, 29], and can also be assessed via visual read [19]. The spread of Aβ pathology has been neuropathologically characterized in terms of Thal phases, and Aβ PET analysis methods have been developed to recapitulate this in vivo [30, 31]. While Thal phase 1 represents amyloid deposits anywhere within the neocortex, Aβ PET imaging has revealed additional granularity in the sequence of brain regional involvement in early amyloid accumulation, indicating that medial and lateral frontal and parietal regions become elevated first [32, 33] (over and above anatomically discordant age-related increases [34]). Thus, while in symptomatic disease, the salient information in Aβ PET scans is generally captured well by a global summary value (see also the “Cross-tracer comparability: Centiloids and amyloid load” section), secondary prevention trials targeting healthy individuals in the earliest stages of amyloid accumulation may focus on these early-enhancing regions, or leverage imaging-based amyloid staging schemes that distinguish these early changes.

In contrast, tau pathology in AD is characterized by a more readily apparent pattern of spread, originating in the entorhinal cortex with additional involvement of neocortical regions in the temporal lobe, association cortices, and finally the primary sensory cortices [35]. Despite inter-individual variations, this well-defined anatomical progression has been systematized in neuropathological staging schemes such as those proposed by Braak [36] or Delacourte [37], and recapitulated in PET imaging studies with recently developed tau PET tracers [38–40]. While there is a general trend for more advanced clinical disease to be associated with higher tau stages, a range of tau stages is generally observed at each clinical stage, possibly reflecting a role of co-pathologies and/or cognitive reserve [7].

As PET tracers for other protein deposits are developed, similar staging approaches can be developed and assessed. Existing neuropathological frameworks as described above for tau provide one starting point; as sufficient data become available, data-driven staging patterns may also be determined from the image features directly [41].

#### Enrichment

The idea of an enriched population is that it has a higher proportion of some desired characteristic, such as risk for rapid progression or homogeneity of the enrolled cohort. However, an important distinction is whether the enrichment is based on biological considerations, such as the presence of the biological profile considered necessary to benefit from the treatment mechanism being tested, or statistical considerations, to simply yield a more efficient clinical trial. The selection of patients based on molecular markers using PET or SPECT as described above may correspond to both. For example, in AD trials, enrolling participants with confirmed brain amyloid by PET enriches for faster progression [42, 43], and baseline measures of tau PET load are also associated with faster progression [44]. If amyloid or tau pathology is, respectively, the target mechanism of action, then the enrichment is also a biological one. Although these terms may have some overlap, biological enrichment is often more synonymous with the concept of personalized medicine, discussed further in the “Personalized medicine” section.

In another example, enrolling early PD participants with reduced striatal dopamine transporter binding on DaTscan SPECT results in a more homogeneous, rapidly progressing population [45] and has been qualified for this purpose by both the European Medicines Agency and US Food and Drug Administration [46]. The concept of enrichment can also refer to the use of less molecularly-specific markers such as those based on clinical or genetic features, or structural imaging via vMRI. One example of this latter case is the use of low hippocampal volume as an enrichment tool for subjects with mild cognitive impairment considered prodromal for Alzheimer’s disease. Selecting subjects with smaller hippocampal volumes results in a cohort that progresses, on average, more rapidly and with reduced variability on clinical outcomes. This approach was qualified for this purpose by the European Medicines Agency in 2014 [47] and is effective over a wide range of cut-points [48] and whether or not the population is confirmed amyloid-positive [49]. However, we note that this approach is essentially a statistical one regarding the detection of slowing of disease progression, with no guarantee that the faster progressors are more likely to be biologically responsive to the treatment. Rather, it makes it easier to detect an effect, if present, and other things being equal. There is also an ethical rationale that exposing an individual to a treatment with possible side effects (and cost to the sponsor and—if/when approved—to the healthcare system) is unwarranted if that individual is unlikely to worsen in the absence of treatment.

An alternative to enrichment for purely statistical considerations (such as rate of progression) is to cast a wider net in terms of inclusion but account for the prognostic (enrichment) variable in the statistical analysis. A good example of this is the relationship between an individual’s age and CAG repeat length (sometimes combined as a disease burden score) and rate of disease progression in Huntington’s disease. While a clinical trial’s inclusion criteria may include some bounds on these variables, allowing a broad range and adjusting for these variables in the statistical analysis allows an optimization of statistical sensitivity whilst maintaining a wide range of people who might benefit from the treatment, if effective [50].

#### Radiological Inclusion or Exclusion

In addition to quantitative and molecular imaging described above, there is also utility in radiological assessments in the trial screening phase. Expert visual review of MRI scans (e.g., T1-weighted, T2-weighted, diffusion-weighted MRI) is commonly used to exclude potentially confounding causes for the clinical phenotype (space-filling tumors, evidence of stroke, etc.). Visual rating scales can be used to index the degree of regional brain atrophy [51, 52] and white matter hyperintensities (associated with cerebrovascular disease) [53].

Occasionally, a neurological disorder has specific MRI-visible signs that help for inclusion. For example, in multiple system atrophy (MSA), the presence of the hyperintense putaminal rim sign on T2-weighted images and hot cross bun sign on T1-weighted images, along with visually assessed atrophy of the putamen, cerebellum and/or the middle cerebellar peduncles, can help support clinical diagnosis of MSA, especially to distinguish it from other movement disorders such as PD, CBD, and PSP [54–56].

#### Personalized Medicine

Many of the imaging approaches reviewed above can be considered as potential tools to identify “the right patient” to receive a given therapeutic. This determination comprises both biological and practical aspects—the individual should not only have the biological perturbation that the therapeutic seeks to ameliorate (not always obvious from clinical symptoms alone) but should also be able to expect a clinically meaningful response with no or with well-circumscribed side effects.

However, while imaging methods can be used for this purpose in drug development, they have an associated monetary cost, patient burden (additional clinic visit, radiation exposure), and often complexity that requires a specialized infrastructure and expertise for both acquisition and analysis/interpretation. The development of a personalized medicine approach conducted as part of the clinical development of a therapeutic may thus seek to identify proxies for advanced imaging tools that will be more easily adopted after the drug is approved. Alternatives such as polygenic risk scores or fluid biomarkers that can be generated from a simple blood test (for example) are examples of companion diagnostics that are more likely to be more acceptable to health systems. In this case, the role of imaging is to help generate a strong scientific evidence base in the drug development phases, rather than serving as the companion diagnostic itself.

There are cases where specialist imaging tools can and are used for diagnostic purposes in clinical practice (for example, [^123^I]ioflupane (DaTscan) for striatal dopamine deficit in movement disorders) and others where this has been proposed (for example, amyloid PET for Alzheimer’s disease) but these require substantial infrastructure and may be supplanted by cheaper proxies (for example, a blood test for amyloid β) as and when they become available.

### Imaging as a Tool to Demonstrate Biodistribution, Target Engagement, and Pharmacodynamic Activity

#### Biodistribution: A New Frontier to Maximize the Value of Novel Large-Molecule Treatment Modalities

The advent of increasing numbers of biologic-based therapeutics and novel molecular modalities in drug development programs (e.g., antibodies, oligonucleotides, peptides, gene therapies) has brought the need to quantify the amount and regional distribution of a novel compound’s exposure in the CNS into sharp focus, as brain penetration is reduced and potentially less homogeneous than that associated with systemically administered small molecules that pass readily through the blood–brain barrier. Radiolabeling these large molecules directly allows the time course and anatomical distribution to be mapped using PET or SPECT imaging [57]. The radionuclide is selected such that its half-life matches the pharmacokinetics of the molecule of interest—so, for biologics, radionuclides such as ^64^Cu (PET, half-life 12.7 h), ^89^Zr (PET, half-life 3.3d), ^123^I (SPECT, half-life 13.2 h), or ^124^I (PET, half-life 4.2d) may be appropriate choices [58].

Although a relatively new area of imaging, this approach enables the anatomical distribution of exposure to the therapeutic to be determined directly, and confirmation of distribution to brain structures of particular interest (a function of the disease and mechanism of action) to be obtained in vivo. Such microdosing studies would usually be performed in small numbers of individuals in the earliest phase of clinical drug development.

#### Target Engagement: Receptor-Occupancy PET Studies

For small molecule therapeutics, engagement of the molecule at its targeted molecular site of action can be measured directly by competitive binding PET studies to assess the dependence of target occupancy on its dose and pharmacokinetic parameters. If a suitable radiotracer is not already available (e.g., for novel targets), then a tracer discovery campaign is usually embarked upon in parallel with the preclinical development of the therapeutic. This approach enables dose-occupancy (and exposure-occupancy) relationships to be determined with excellent sensitivity at peak and trough PK exposures, allowing an informed selection of dose and dosing schedule for subsequent trials [59]. For small molecule programs in neuroscience, this has been one of the most successful applications of imaging to CNS drug development. It has been particularly prevalent in psychiatry programs [60, 61] but is also applicable to neurological disorders in the case of small molecule therapeutics with a stable binding target [62].

#### Pharmacodynamics

The term pharmacodynamics refers to a measurable pharmacological effect of a compound on some aspect of the body’s physiology. Target engagement per se does not guarantee a relevant pharmacodynamic effect. Both will be dose-dependent, and the level of target engagement required to achieve a given magnitude of pharmacodynamic effect is dependent on the pharmacological mechanism and nature of the readout. The term pharmacodynamic is sometimes used quite broadly to encompass disease modification (e.g., slowing of brain atrophy or of aggregate accumulation; discussed further below) and could conceivably cover side effects as well, but in drug development it is often used in a more focused way to refer to changes that are biologically proximal to the mechanism of action of the intervention, that ideally can be measured in a small, early-phase trial and de-risk subsequent development [63].

An example of an imaging marker to potentially detect a pharmacodynamic effect in Huntington’s disease is phosphodiesterase 10 (PDE10) PET, a measure of medium-spiny neuron density in the striatum. Clinical studies have shown that striatal PDE10 PET binding potential correlates well with the burden of pathology and has promising longitudinal change characteristics, but might be most sensitive early in the course of disease [64–69]. Use of PDE10 PET to detect a treatment effect in clinical drug development has yet to be reported, but preclinical studies suggest that it may be relevant for certain treatment mechanisms [70].

With increasing understanding of the role of neuroinflammation in various neurological disorders, therapeutics targeting these processes are increasingly of interest and PET probes of neuroinflammation could also provide key markers of a pharmacodynamic effect. At the present time, PET tracers targeting the 18 kDa translocator protein (TSPO) have been the most widely studied, providing in vivo evidence of increased TSPO expression in many neurological conditions [71–78]. TSPO tracers suffer from two drawbacks. One is that a TSPO Ala147Thr polymorphism rs6971 results in different binding affinities of second-generation TSPO PET tracers in different individuals, with only the medium (C/T heterozygote) and high (C/C homozygote) binders yielding tractable PET signal [79]; this does not however preclude hypothesis-driven studies in those subsets of the population. (In contrast, first-generation TSPO tracers such as [^11^C]PK11195 suffer from poor specificity and signal-to-noise ratio.) The other is that TSPO is considered a relatively non-specific target, not directly reflecting more specific modulation of neuroinflammatory processes that is the therapeutic target. An alternative neuroinflammation readout is provided by PET tracers binding to monoamine oxidase B (MAO-B) in astrocytes, which has indicated increased astrocytosis in early stages of Alzheimer’s disease and Parkinson’s disease [80–83]. PET tracers for a number of other neuroinflammation-related molecular targets have been developed [84–86], but this remains a very active area of current imaging research.

MRI-based pharmacodynamic markers in neurology primarily reflect brain function. Task-based functional MRI (fMRI) is challenging in neurological disorders due to the cognitive and motor impairments and the lack of standardization of the ancillary equipment needed to acquire the data. There is little convincing evidence of its utility as a biomarker in these diseases. Resting state fMRI (rsfMRI) has been far more widely studied due to the fact that ancillary equipment and having participants perform specific tasks in the scanner is not required but, here too, strong and reproducible evidence of its utility remains to be demonstrated. Arterial spin labeling (ASL) measures of resting cerebral blood flow have begun to be investigated but clear evidence of disease signatures and longitudinal performance characteristics remain to be fully elucidated. There are also practical challenges in terms of the availability and comparability of sequences at different sites and scanners. Recent availability of product 3D ASL sequences from several scanner manufacturers may help the further investigation of blood flow biomarkers across neurological disorders.

### Imaging to Provide Evidence of Disease Modification

For therapeutic approaches aiming to modify (slow or halt) the course of disease, it is critical to have plausible biomarker evidence that this is the case. In neurology trials, there is a large role for disease-related imaging markers to help provide this evidence. Markers for this purpose should possess both face validity (e.g., clear relationship to the disease at hand) and construct validity (e.g., adequate technical performance characteristics and quantitative relationship to clinical variables, for sample sizes and time spans relevant for clinical trials) [87] (Table 1).

**Table 1:** Checklist of technical and evidentiary standards to help delineate the utility of disease-related biomarkers

Checklist of technical and evidentiary standards	
Technical standards	
1	Are acquisition and analysis protocols standardized? • Are there recommended or minumum standards? • What is the sensitivity of the marker to variations in acquisition parameters? • Are there recommended or minimum standards?
2	What is the test–retest variability of the marker?
3	Are normative reference ranges established?
Evidentiary standards	
Face validity	
4	How does the marker relate to the underlying biology of the disease?
5	Is the marker clearly different in the disease state compared with matched healthy controls?
6	How does the marker relate cross-sectionally to clinical scales and disease or symptom severity? • Is the relationship monotonic?
Construct validity	
7	What are the longitudinal change characteristics of the marker? • E.g., Cohen’s d effect size or similar • How does this depend on disease severity?
8	Does the baseline value of the marker predict subsequent clinical change? • On what time scale?
9	Does longitudinal change in the marker correlate with concurrent longitudinal change in clinical scales? • Does this hold on time scales typical of clinical trials? • Does longitudinal change in the marker over a relatively short period predict subsequent longitudinal change in clinical scales or other biomarkers over a longer period?
10	Are there data with the marker from prior interventional trials? • Is the technical performance of the marker maintained in the clinical trial context? • What is the background landscape of treatment effects from previous trials? • Are the relationships between the marker and clinical scales maintained in the presence of treatment?

The concept of disease-related outcome biomarkers has some overlap with pharmacodynamic biomarkers but tends to refer more specifically to measurands related more closely to the disease itself rather than the specific mechanism of action of the treatment. The presence of amyloid β and tau aggregates is a defining characteristic of Alzheimer’s disease and both of these can be detected in vivo using PET tracers. For therapeutic mechanisms targeting these pathologies, they thus represent both pharmacodynamic and disease-related markers. A number of therapeutic trials have targeted amyloid pathology and slowing of accumulation [88] or reduction of aggregated amyloid levels below baseline values [89–91] provides evidence the disease biology is being modified by the therapeutic candidate. The recent advent of tau PET tracers [92–99], exhibiting a closer anatomical and temporal relationship to clinical symptoms than amyloid PET [100], has been an enabling feature for therapeutic programs targeting tau pathology and promises to provide another important window on how novel potential therapies interact with the Alzheimer’s disease process in the living brain [101]. The development of PET tracers specific for protein aggregates underlying other neurological disorders will be similarly transformative for clinical drug development in those areas.

Less target-specific imaging methods also have considerable value as more “treatment agnostic” markers. The best-established examples of this are disease-specific patterns of brain atrophy from vMRI [102–113] or altered glucose metabolism from FDG-PET [114–119]. These markers are typically relatively well-correlated with clinical outcomes, both in terms of natural history data as well as treatment response, although the strength of the association and anatomical regions implicated may depend on disease severity and the nature of the clinical scale [106, 107, 120–123]. Recent progress toward PET tracers more specifically reflecting synaptic density is a potential future improvement in this context [124, 125].

Such markers also typically require larger and longer trials to be adequately powered, compared with a pharmacodynamic markers reflecting biology more proximal to the mechanism of action. Some imaging markers can sit in either category, with their context of use in early- or late-phase trials largely dependent on the magnitude of treatment effect—for example, the removal of amyloid plaques below baseline levels in AD can be detected in relatively small trials and represents a clear pharmacodynamic effect (although also disease-related), confirming the hypothesized mechanism of action and supporting continued clinical development [89, 90]. More subtle effects on amyloid removal require larger trials to detect and, although also arguably a pharmacodynamic effect, would need to be tested in larger trials [4, 88].

MRI measurement of gadolinium contrast-enhancing lesions on T1w scans and new or enlarging lesions on T2 scans (lesion count or total volume) is well-established as a disease-related biomarker in relapsing–remitting multiple sclerosis [126] with overall treatment effects on lesion load correlating well with treatment effects on clinical outcomes in phase 2 and 3 trials [127]. This is one of the best examples of an imaging marker having demonstrable predictive utility in a phase 2 scenario (to reduce the risk of failure in phase 3) and as a demonstration of biological activity in phase 3.

### Imaging as a Means of Monitoring Drug Safety

MRI is often used to monitor for potential CNS effects indicative of side effects. Most typically, it would be used for this purpose in the early clinical phases of drug development, to (hopefully) demonstrate the absence of concerning radiological findings and thus de-risk the molecule for further development. Safety monitoring in such cases can then be less extensive in larger, late-phase trials. On the other hand, if a clear safety finding related to the molecule’s pharmacology is expected or discovered, large-scale safety monitoring may also be needed in later-phase trials. A well-known example from AD is monitoring for amyloid-related imaging abnormalities (ARIA) [5, 12, 90, 128, 129].

## Standardization of Imaging in Clinical Trials

Standardization of multi-site imaging in clinical trials starts with the engagement of a centralized imaging core lab tasked with managing all aspects of imaging for the trial. This includes evaluation and training of sites, harmonization and site-by-site implementation of acquisition procedures, quality control of imaging data and remediation of issues with sites, management of radiological reads, and performing centralized quantitative analyses. All this needs to be managed with data handling and computer systems that are regulatory (e.g., CFR21.11) compliant and maintain access controls and audit trails.

In addition to standardization of imaging across sites within a given trial, it is also relevant to consider standardization across trials. Established best practices are extremely useful and facilitate the comparison of results from different trials. Moreover, regulators tend to look kindly on the use of standardized methods, as reflecting a degree of maturity in the methodology and consensus in the field.

In the following we consider some specific aspects pertaining to PET and MRI, respectively.

### PET

#### Standardization of Dosing and Image Reconstruction

For multisite PET studies, it is highly likely that different camera models will be used. It is important to harmonize the reconstruction and attenuation/scatter correction methods to the extent possible, with the aim of maximizing comparability of resulting scans from different sites. Often, a test scan with a test object (e.g., a Hoffman phantom) will be requested from each site, in order to check adherence to the scan and reconstruction instructions and potentially to calibrate the camera-specific point-spread function. The positioning of the acquired image FOV should be consistent across participants and visits, avoiding portions of the superior cortex or inferior cerebellum being cut off, for example. The centralized image processing may smooth all images to the same effective resolution, taking into account the intrinsic smoothing resulting from each camera.

The targeted and maximum injected radiation dose and allowable variation should be specified in the study protocol and monitored during the study. It is uncommon for multisite PET studies to require blood sampling to aid modeling and quantitation of the image data, but if this is the case, these procedures and the analysis of the samples to derive radiotracer and radiometabolite concentrations must also be standardized and monitored.

Standardization of PET image analysis across studies and across different tracers for the same target (e.g., amyloid β) has been facilitated by recent developments to harmonize how images are processed and quantified (see “Interpretation of imaging in clinical trials” section).

#### Static Scan Protocols

For multisite PET studies in patient populations, the scanning protocol is typically a “static” scan, covering a relatively short time window starting some time after the injection of the radiotracer (e.g., a 20-min scan starting 60 min after tracer injection—the precise timing depends on the nature of the tracer and the needs of the study). Often, such scans are split into several, shorter, consecutive frames to allow correction for head motion. Such scans are usually expressed in standardized uptake value (SUV) units, being the ratio of the reconstructed counts in each voxel multiplied by the body weight of the subject and divided by the injected radiation dose, and further quantified in terms of a SUV ratio (SUVR), being the ratio of the signal in target regions of interest (assumed to reflect specific binding) to that in a reference region (assumed to reflect non-specific binding only) from the same image. This paradigm has been widely used for amyloid and tau PET scans in Alzheimer’s disease.

**Fig. 2:**
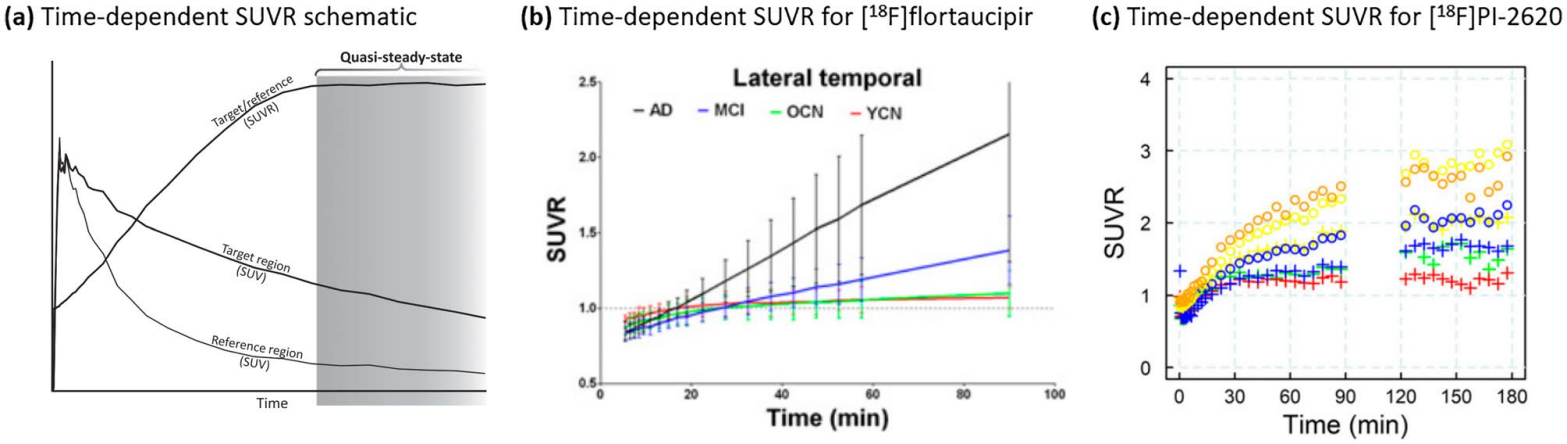
Time-dependent SUVR plots provide a means to assess the temporal stability of SUVR measurements, can help identify an optimal static scanning window, and can highlight instances where deviations in acquisition time might contribute to additional variability. (**a**) Schematic showing the ideal case where a quasi-steady-state of SUVR(t) is obtained at a certain time post-injection. (**b**) Average SUVR(t) curves across small cohorts of subjects at different disease stage for [^18^F]flortaucipir in the lateral temporal lobe, indicating that a quasi-steady-state is not achieved (on average) in more advanced disease stages. (**c**) SUVR(t) curves from a single Alzheimer’s disease individual using [^18^F]PI-2620, where each color represents a different brain region, indicating that a quasi-steady-state is not achieved in regions with higher tau burden (**a** was originally published in the Journal of Cerebral Blood Flow and Metabolism [130]© SAGE Publishing; **b** was originally published in the Journal of Nuclear Medicine [131] © SNMMI; **c** was originally published in the Journal of Nuclear Medicine [96] © SNMMI)

Scans of this type minimize patient burden and scan time and cost but implicitly assume that the radiotracer has reached an approximate equilibrium in the brain between the kinetics in the target and non-specific compartments. (In other words, if the SUVR in the target region(s) is calculated as a function of time since injection, this SUVR(t) curve has reached a plateau during the static scan acquisition window [130] (Fig. 2a). Indeed, such time-resolved SUVR plots are informative to help determine the optimal acquisition window [96, 131–133].) In practice, however, many PET tracers for which static scans are employed for practical reasons do not always exhibit a perfectly stable SUVR signal during (and either side of) the scanning window, a property often exacerbated in regions of high binding [131, 133] (Fig. 2b, c). This means that variations in the timing of the acquisition window relative to tracer injection can result in a substantial increase in variability of the final calculated SUVR values. This can be particularly problematic for longitudinal scans, where inaccuracies in scan timing can manifest as artefactual changes in signal intensity. It is thus critical that the scan timing, relative to tracer injection, is kept constant across sites and especially within-subject for longitudinal studies.

#### Dynamic Scan Protocols

The gold standard for PET quantification is a full kinetic analysis of regional time-activity curves of detected radioactive counts in the brain [134]. However, this is a specialized endeavor in terms of both image acquisition and analysis. Depending on the tracer, the scan itself can take 2–3 h, and an arterial line may be needed to quantify circulating radiotracer levels to derive an input function for the kinetic modeling. This approach is common in single-site, early-phase, trials but highly demanding to generalize to a multi-site context. If a tracer is planned to be more widely implemented, a close association between an appropriate full kinetic analysis and an SUVR approximation should first be demonstrated [96, 131, 135].

A compromise that is sometimes implemented is a “coffee break” protocol, in which the subject is scanned (without an arterial line) for, say, the first 20–30 min after injection, and then again at a later time, say, 60–90 min after injection [136]. These “early frames” and “late frames” scans can be combined in an analysis that captures both the early, highly dynamic tracer kinetics as well as the later signal reflecting specific tracer retention. These data allow kinetic modeling (with certain assumptions), whilst reducing patient burden compared with a full kinetic scan protocol.

For some tracers, the early frames signal also provides a signal reflecting cerebral blood flow, which is of increasing interest as an outcome measure of itself, thought to represent a potential biomarker of neurodegeneration. This can be quantified via a kinetic analysis (R1) or from a simplified SUVR-like “static” analysis of the first few minutes and has been shown in AD to manifest patterns of hypoperfusion in the brain that closely mimic patterns of hypometabolism obtained from FDG-PET [137–140]. Here too, it is critical to monitor image acquisition such that the start of image acquisition is timed as specified and consistently with respect to tracer injection.

#### Standardized Reporting of PET Imaging Results

Consensus guidelines for standardized and complete reporting of PET imaging study results have recently been published [141]. These represent best practices for data reporting and sharing and are aimed at maximizing reproducibility, transparency, and the potential for data pooling. While most straightforwardly applicable to academic studies, they also serve as useful guidelines for publications of industry studies and in the event of data sharing of clinical trial images, as is becoming increasingly common via organizations such as the Critical Path Institute.

### MRI

#### Standardization of Image Acquisition

In a multi-site trial, the MR equipment (including scanner model and bore length, field strength, software version, head coil, gradient capabilities and availability of research sequences) will in many cases be different between sites. It is thus critical to minimize variability at point of acquisition by standardizing the choice of equipment (e.g., only 3-T scanners accepted), and harmonizing the specific sequence acquisition parameters to the extent possible, across scanners and sites. In practice, and especially in larger trials, this will be a compromise with some residual variability in acquisition unable to be resolved. It is also important to maintain consistent positioning of study participants in the scanner bore, for example by centering the positioning laser on the nasion, to avoid image distortion due to B_0_ field inhomogeneities that arise away from the scanner isocenter. Such distortions can be particularly problematic on short-bore scanners, in which these inhomogeneities are more extreme.

Head motion is a common issue in neurology trials, and a common source of image quality control failure. This should be minimized at the point of acquisition by ensuring the research participant is comfortable and that their head is firmly restrained within the head coil, and that they understand the need to keep still. The tolerance for head motion is lower for quantitative analyses compared with standard radiological assessment and so the trial sites should be trained on motion minimization and monitored by the imaging core laboratory throughout the trial. Retraining of the site by the core laboratory may be necessary if head motion is particularly prevalent at a given site. This issue emphasizes the need for continuous, real-time oversight and quality control of the images as they are acquired during the trial.

If possible, it is advisable to include two back-to-back acquisitions of the same sequence in the MR protocol, if the sequence acquisition time is sufficiently short and if that sequence provides the primary MR outcome for the trial. The rationale for this is that if one of the scans has quality issues (e.g., due to motion), then the other one may be usable, reducing the overall rate of missing data in the study. A common example is vMRI, where back-to-back 3DT1 sequences are often acquired in neurology trials. This has been facilitated by the advent of accelerated sequences, reducing the scan time and allowing two 3DT1 scans to be obtained in approximately the same time previously required for one. This approach is however contingent on demonstration that accelerated acquisitions yield comparable quantitative outcomes to unaccelerated scans—this has been demonstrated in the case of Alzheimer’s disease [142].

#### Standardization of Image Analysis

Standardization of MR image analysis across different trials, sponsors and core imaging laboratories needs to be compatible with the use of proprietary image processing algorithms and with the continual technical advances in image analysis methodologies. One way to achieve this is to achieve consensus agreement on the measurand, rather than the means by which it is measured. For example, in the case of vMRI, standardized definitions of key brain structures, in terms of anatomical landmarks and detectable MR image contrast, enables different algorithms to be optimized for the segmentation of the same brain structure.

The flagship example of this to date has been the harmonized hippocampus project [143, 144]. Prior to this effort, different automated segmentation algorithms often generated widely different values of the hippocampal volume from the same image, simply because they were trained on different anatomical definitions of what constituted the hippocampus. The harmonized protocol project convened a panel of experts in manual tracing of the hippocampus images and used a multi-round Delphi panel framework to determine agreement on the MR anatomical boundaries of the hippocampus. This harmonized protocol was then applied to a set of reference images from the ADNI study, providing a freely available “gold standard” set of anatomical masks against which automated algorithms can be trained and evaluated. Similar efforts could be valuable for other brain regions in other diseases, for example the caudate or putamen in Huntington’s disease.

The availability of standardized definitions of brain structures in this way then allows for the definition of universal cutoff values that are independent of the specific algorithm used to generate them. Age and intracranial volume (ICV) are key confounding factors that also need to be corrected for, and other variables can also impact the resulting values [145, 146]. Correction for ICV may be achieved by establishing a regression relationship in a control population, or simply by expressing volumes as a fraction of ICV. In the absence of harmonized volumetric definitions (which remains the case for most brain structures and ICV), values can be referenced to a reference control population, analyzed using the same algorithm(s), and expressed as percentiles or z-scores with respect to the normal range [48, 147, 148]. Such results are often presented visually in the form of deviations from age-dependent curves of normal atrophy. In this way, cutoff values expressed in terms of percentiles or z-scores referenced to a control population can partially overcome algorithm-dependent differences in raw volumes and result in comparable performance, for example in the case of enriching a mild cognitive impairment population for fast progression to Alzheimer’s disease [47, 48]. However, different anatomical definitions between different software packages and atlases remain an important variable to be evaluated for its impact, for brain structures where a consensus agreement on segmentation boundaries has not yet been established.

## Interpretation of Imaging in Clinical Trials

### PET

#### Cross-Tracer Comparability: Centiloids and Amyloid Load

One of the issues with interpreting PET results is interpreting the quantified outcome measures across different tracers for the same target, and across different clinical trials for which different processing pipelines may have been used. For a given tracer, the final quantified numbers (e.g., SUVR or DVR) and associated key values (e.g., a positivity cutoff threshold) depend on details of the image analysis, including the spatial pre-processing steps, whether gray matter masking or partial volume correction were used, and the choice of reference and target regions. Even if these are all kept constant, different tracers for the same target will yield different values. Some way to standardize the units in which quantified PET data are reported is thus very important for a more widespread and easily understood use of these tools.

**Fig. 3:**
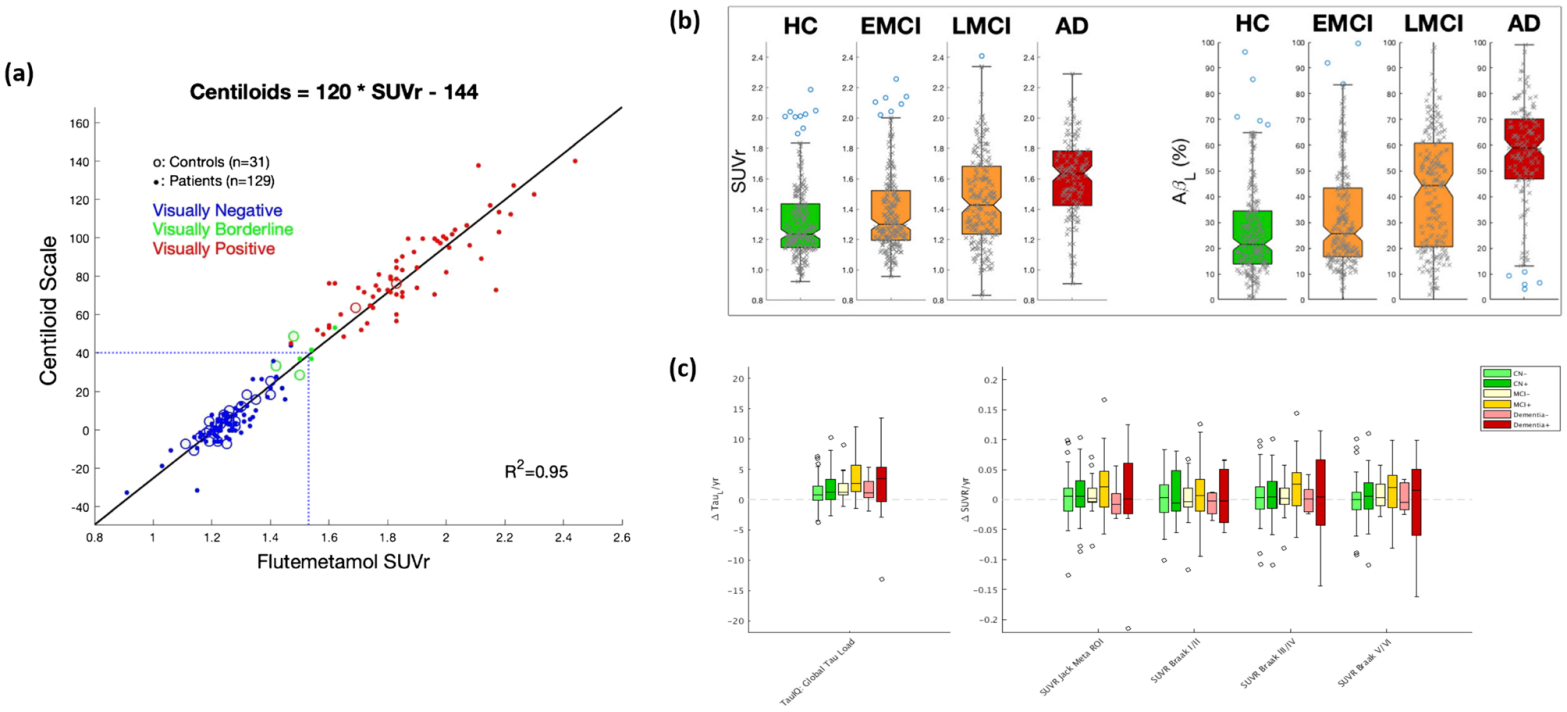
(**a**) Mapping of [^18^F]flutemetemol amyloid PET SUVR values (*x*-axis) into Centiloids (*y*-axis) [158]. (**b**) Side-by-Side comparison of [^18^F]florbetapir SUVR values and Aβ_L_ values across Alzheimer’s disease stages from the ADNI database [160]. Note that Aβ_L_ values have well-defined floor and ceiling levels. (**c**) Side-by-Side comparison of longitudinal change in global TauL and regional SUVR values from [^18^F]flortaucipir scans in the ADNI database (**a** was originally published in the European Journal of Nuclear Medicine and Molecular Imaging [158], reproduced under the terms of the Creative Commons Attribution 4.0 International License (http://creativecommons.org/licenses/by/4.0/); **b** was originally published in the Journal of Nuclear Medicine [160] © SNMMI; **c** was originally published in the Journal of Nuclear Medicine [162] © SNMMI; axis labels have been redrawn for legibility)

Two recent efforts have attempted to address this issue for amyloid PET tracers. The first of these, known as “Centiloids,” specified a standardized processing pipeline for [^11^C]-PiB as a reference tracer, along with a method to scale any other amyloid tracer image analysis to the same scale [149, 150]. In this approach, the Centiloid units are anchored to values of 0 and 100, with 0 being defined as the average value in young healthy controls, and 100 defined as the average value in typical patients with Alzheimer’s disease dementia (Fig. 3a). Regression equations mapping individual tracer and processing pipeline combinations to Centiloid units have been determined for a number of amyloid tracers [151–155]. This has enhanced inter-tracer comparisons, facilitated research into tracer-independent positivity thresholds [156–158], and improved the interpretability of clinical trial results showing effects of anti-amyloid treatment on imaging data [91], although some residual variability remains [150, 159].

The second such approach, known as amyloid load (Aβ_L_), calculates a global, whole-brain amyloid burden using an algorithmic method known as Amyloid^IQ^ [160]. In this approach, an individual amyloid PET image is fit as a linear combination of a canonical non-specific binding image and a canonical “carrying capacity” image, with the coefficient weight of the latter equating to Aβ_L_ [161]. The method is scaled such that the values of Aβ_L_ range from 0% for a scan with no amyloid to 100% for a scan with the highest level of amyloid (Fig. 3b). This method was originally developed for [^18^F]florbetapir but has since been generalized to other amyloid tracers. Similar to Centiloids, this approach facilitates inter-tracer and inter-trial comparisons of amyloid PET data, the expression of amyloid positivity cut-off values and is expressed on an intuitive 0–100 scale of units. However, whereas 100 on the Centiloids scale reflects an average AD amyloid load, 100 on the Aβ_L_ scale reflects the maximum load. This scale may thus be prone to a ceiling effect in individuals with near-maximal amyloid load, in scenarios where a slowing of accumulation is expected. Moreover, neither the Centiloid nor Aβ_L_ scales may be optimally sensitive in prevention studies where a more subtle slowing of early (regional) amyloid accumulation is sought.

Both the above approaches are being extended to tau PET tracers [162] (Fig. 3c), and the load/IQ method is also being developed for DaTscan in the context of Parkinson’s disease. Other cross-tracer harmonization methods have also been proposed [163, 164]. While amyloid and tau PET have been the initial focus of these approaches due to their widespread use in AD research, similar harmonization efforts could be performed for other tracers and molecular targets as the imaging tools and data become available.

#### Qualitative Changes in Status with Treatment: Positive to Negative?

Many current therapeutic programs aim to ameliorate neurological disorders by targeting the misfolded protein aggregates that are their neuropathological hallmarks. While in some cases the expectation is that the treatment will slow or halt the formation of new aggregates, in others, the load of such deposits in the brain can be drastically reduced to levels below the pretreatment baseline. Such clearance of amyloid plaques or tau tangles has been strikingly demonstrated in animal models [165, 166] and can be detected in humans when PET tracers that bind to the protein aggregate in question are available—currently limited to amyloid and tau in AD.

Some recent anti-amyloid treatments have demonstrated in clinical trials that amyloid load as detected by PET can be reduced to levels far below baseline in many trial participants, and in some cases reduced below the level used as a threshold for amyloid positivity [90]. In other words, certain individuals have qualitatively changed status from “amyloid positive” to “amyloid negative.” According to recent research criteria for AD [15, 16, 167], in which amyloid positivity is a defining characteristic of AD from the earliest pre-symptomatic stages, one interpretation of these changes is that these individuals no longer have AD. This qualitative change in state per se thus provides an alternative outcome, with implications about interpretation of the biomarker state of the participants, as a complement to statistical analyses of quantitative variables (e.g., SUVR or other continuous measures of radiotracer binding).

#### Spread of Pathology: Local and Distributed Changes

Therapeutics seeking to slow or stop the spread of templated proteinopathies, hypothesized to proceed in a prion-like way [168], are of increasing interest for drug development in neurology [169]. Changes in the anatomical distribution of protein aggregates can be tracked longitudinally in vivo when appropriate PET tracers are available, albeit with less sensitivity than neuropathological examination [170].

In Alzheimer’s disease, the development of amyloid plaque load in the brain follows an anatomical sequence that has been codified neuropathologically [171] and can be replicated in vivo using amyloid PET tracers [31, 32] but, since a broad cortical distribution occurs early in this process, and well before the onset of clinical symptoms [14], global measures of PET amyloid load are still typically used, although an early accumulating brain region may be more sensitive in secondary prevention studies. In contrast, the anatomical distribution of tau tangles follows a more dynamic change over the preclinical, prodromal and symptomatic phases of the disease, spreading from medial temporal regions to broader involvement of temporal and association cortices, and with primary sensory areas affected last of all [35–37]. These anatomical stages are recapitulated well in vivo using tau PET tracers [38–40, 172], opening the way for therapeutic effects on tau pathology in the brain to be represented in terms of changes in tau spreading. For a given scan, this concept may be operationalized as an index of spread or as a pattern of binding intensity in brain regions representative of different tau stages, as alternate outcomes complementary to more global measures of tau burden. Indeed, recent analyses have indicated that longitudinal changes in tau PET patterns are related to inter-regional brain connections [173, 174]. However, it remains to be determined whether staging-based outcome measures will translate into increased sensitivity to detect treatment effects, given substantial inter-individual heterogeneity in the anatomical distribution of tau pathology [175]. Moreover, the presence of local trans-synaptic spreading (below the resolution of PET scans) means that most neocortical brain regions exhibit a continued increase in tau signal as the global signal increases, such that changes in anatomical stage may reflect an “iceberg effect” rather than a phenomenon mechanistically different than that reflected in regional signal intensities. As such, a net reduction in global tau brain burden may manifest as an apparent reduction in tau stage.

Proteinopathies relevant to other neurological disorders are also of increasing interest for drug development. These include α-synuclein for Parkinson’s disease and related synucleinopathies, TDP-43 for amyotrophic lateral sclerosis and frontotemporal dementia, as well as mutant huntingtin in Huntington’s disease and poly-Q aggregates in spinocerebellar ataxias. Some of these already have well-defined patterns of hypothesized spread based on neuropathology studies [176–179]. If and when PET tracers for these other protein deposits become available, the extent to which changes in the PET signal can be quantified and interpreted as a spreading phenomenon or a more traditional level of overall burden can be assessed.

#### Individualized Maps of Abnormality: Z-Scores

Interpretability of images or ROI summary measures from PET tracers such as FDG can be improved by expressing them as z-scores or percentiles relative to a control population. This is because, in contrast to amyloid or tau PET images, where a control population likely has relatively flat images of SUVR ~ 1 and thus any signal elevations are by definition abnormal, images from tracers such as FDG have a region-dependent signal in the absence of disease from which deviations in signal units such as SUVR can be more difficult to interpret. Converting image or regional profiles of FDG-PET binding to z-scores [180] results in individualized fingerprints of abnormal signal that are more intuitive to understand and easier to compare across individuals and across studies. Since there may be some dependence on processing methodologies, the reference population images should be processed using the same analysis pipeline.

This approach is also being increasingly used for other types of imaging, such as volumetric MRI.

### MRI: Contextual Setting and Strategies for Dealing with Pseudo-Atrophy

Disease-specific patterns of brain atrophy are a clear feature of most neurological disorders and represent disease-related biomarkers for which the longitudinal change is expected to be slowed by successful disease modifying treatment. Disorders such as Alzheimer’s disease, Huntington’s disease, behavioral variant frontotemporal dementia, progressive supranuclear palsy, multiple sclerosis, amyotrophic lateral sclerosis, and many ataxias all present with notable brain atrophy measurable on vMRI scans.

The quantified outcomes from vMRI are macroscopic measurements of brain structure, e.g., regional volumes and cortical thicknesses, or image-based measures of change between two scans. Biologically, these volumetric measures sum a variety of microstructural contributions and thus reflect not only the density of neurons but also that of glial and astrocyte cells, cell processes and the extracellular environment. Hence, interventions that differentially alter these different components could potentially lead to unexpected changes in brain atrophy measures. For example, treatment-induced changes in glial activation status or in the amount of edema could potentially confound the interpretation of vMRI changes in treatment trials. This phenomenon is sometimes known as “pseudo-atrophy” and has been most extensively studied in the context of multiple sclerosis, where compounds with anti-inflammatory effects have elicited apparent acceleration of brain volume loss within the first 1–2 years of treatment [181, 182]. These effects have been found to be stronger with higher levels of baseline neuroinflammation, as reflected by the gadolinium enhancing lesion load, and to be driven by reductions in white matter, rather than gray matter, volume [181].

Similar confounding effects may arise in other disorders as well, especially as therapies designed to modulate the brain’s immune pathways become of increasing interest. A number of approaches can help increase the interpretability of treatment effects on vMRI in clinical trials, as summarized in the following. Several of these ideas may also be applicable to other imaging outcomes.

#### Longitudinal Relationships Between Imaging and Clinical Markers

It is important to understand the longitudinal relationship (change vs. change) between the imaging outcome and relevant clinical instruments in the absence of treatment. A strong relationship will underpin the construct validity of a given imaging metric as a disease-related biomarker and inform whether or not a concordant treatment effect on a given clinical outcome is to be expected. Regional topographic correlations between longitudinal change in cognitive tests and longitudinal measures of brain atrophy have been reported in mild cognitive impairment [120] and mild dementia [183] phases of Alzheimer’s disease. Strong relationships between lobar atrophy and concurrent longitudinal change in several global clinical scales have also been reported in variants of frontotemporal dementia [184], and global and regional atrophy rates have been shown to correlate to different degrees with various clinical instruments in Huntington’s disease, with the strongest relationships found overall for a test of executive function and a novel composite outcome measure [122].

However, not all imaging metrics will correlate with all clinical scales, and the relationship will generally be a partial one. More psychometrically specific tests will typically be related to more focal patterns of brain atrophy or of other imaging measures. Relationships identified in natural history data sets should be confirmed in the placebo arm of interventional trials, as an unexpected behavior of the control group can be a potential confound to the interpretation of treatment effects (or lack thereof).

#### The Landscape of Treatment Effects from Previous Trials

**Fig. 4:**
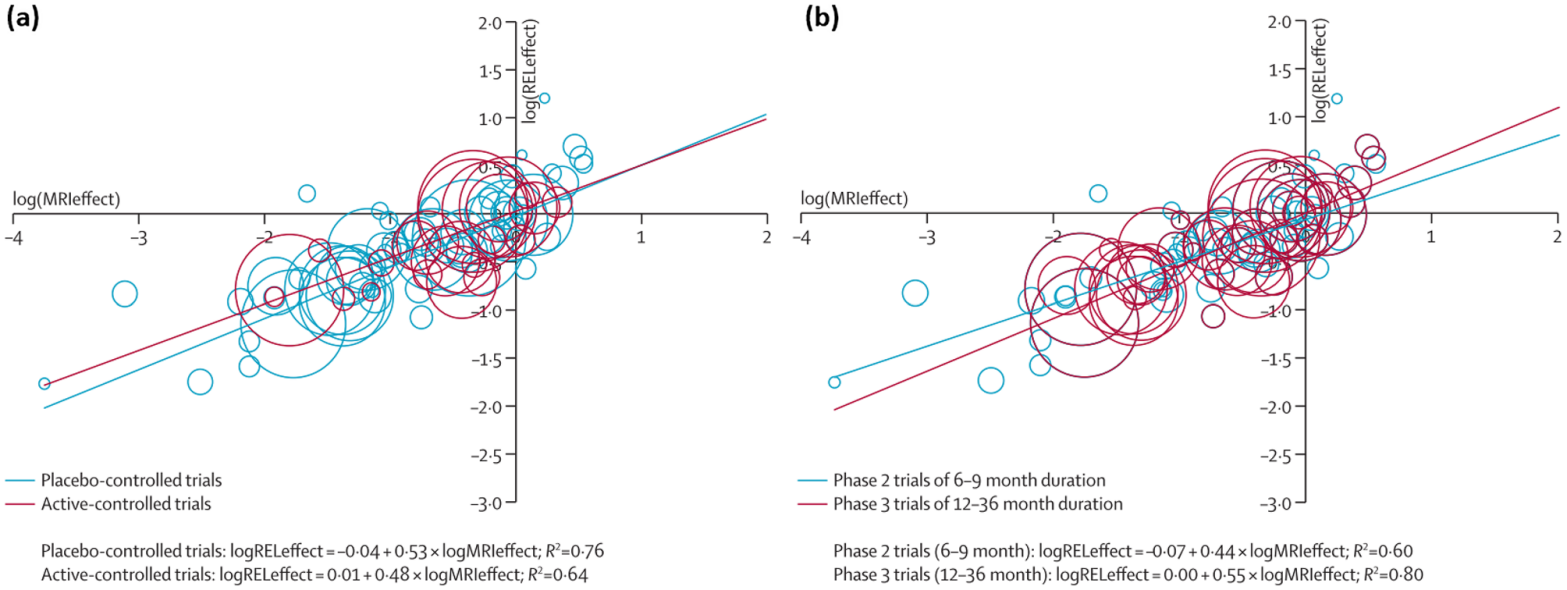
Meta-analysis of reported treatment effects on T2 active lesion load from MRI versus treatment effects on clinical relapses, showing similar relationships for (**a**) placebo-controlled and active-controlled trials, and for (**b**) phase 2 and phase 3 trials. Figure reproduced with permission from [127]

It is useful to be able to interpret the relative magnitude of treatment effects on imaging and clinical outcomes in the context of data from previous treatment trials. Ideally, prior trials have confirmed that treatment effects on the imaging outcome measure are concordant with those on relevant clinical scales. This has been convincingly demonstrated for both enhancing lesion load and brain atrophy in the case of multiple sclerosis [121, 127, 185, 186] (Fig. 4). Such data are important to confirm that imaging-clinical relationships that hold longitudinally in the context of natural history data are maintained in terms of drug effects in interventional trials. It is important to remember that even in well-powered trials, some statistical variability remains, and so these background data provide useful context in which new results can be appropriately interpreted.

#### Graphical Analysis: Disease-Modifying vs. Non-Specific Effects on Brain Atrophy

Notwithstanding the a priori specification of a primary vMRI outcome metric for statistical analysis, analyzing treatment effects across a number of brain regions affected to different degrees by the disease process allows the overall anatomical pattern of atrophy rate changes to be interrogated. A straightforward graphical analysis of the group-level imaging data across the different brain regions can help distinguish whether the treatment effect is more consistent with a modification of the disease process or a non-specific effect.

**Fig. 5:**
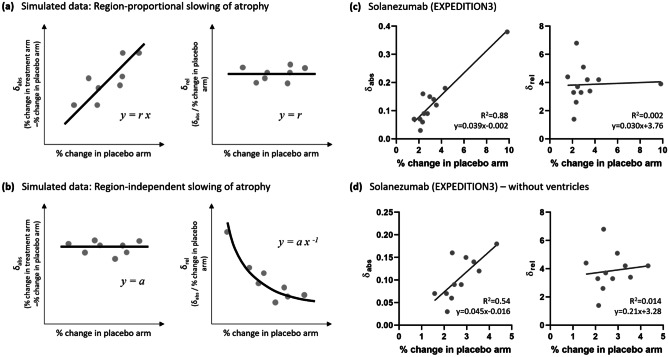
(**a**), (**b**) Theoretical dependence of absolute and relative differences between volumetric changes in treatment and placebo arms on change in the placebo arm for (**a**) the case where the reduction in volume loss is directly proportional to the rate of change in the placebo arm, consistent with a slowing of neurodegeneration, and (**b**) the case where the reduction in volume loss is independent of the rate of change in the placebo arm, such as might result from a non-specific inflammatory effect. Each dot in these ensemble plots represents a different brain region (the data points in (**a**, **b**) are illustrative only). In the case of region-proportional slowing (or acceleration) (**a**), this framework yields three estimates of the relative slowing parameter,* r*. These are the slope of the regression line from the analysis of absolute change, and the average and *y-*intercept from the analysis of relative change. Here, *r* represents the fractional slowing of atrophy in the treatment arm relative to the placebo arm (e.g., *r *= 0.25 would correspond to a 25% slowing). In the case of region-independent slowing (or acceleration) (**b**), this framework yields three estimates of the absolute slowing parameter, *a*. These are the average and *y*-intercept of from the analysis of absolute change, and the coefficient of the inverse relationship from the analysis of relative change. Here, *a* represents the absolute slowing of atrophy in the treatment arm relative to the placebo arm (e.g., *a* = 0.01 would correspond to 1% absolute slowing of brain volume loss). If the treatment arm evidences faster volume loss than the placebo arm, δ_abs_ or δ_rel_ are negative. (**c**, **d**) Ensemble plots and regression analysis for vMRI data reported for vMRI outcome measures from the EXPEDITION3 trial of solanezumab. The regressions indicate that in this case the overall pattern of the treatment effect on brain atrophy is most consistent with the region-proportional scenario (**a**). With all 12 vMRI metrics included (**c**), the three estimates of the relative rate of slowing were 3.9%, 3.8%, and 3.8%, highly consistent, with a coefficient of variance of only 1%. When the ventricles were excluded (**d**), the three estimates were 4.5%, 3.8%, and 3.3%, still consistent, with a coefficient of variance of 13%

Consider how such a pattern of atrophy would be affected by a treatment that modifies these rates of volume loss in two different ways. A plausible disease-modification effect would be expected to alter the rate of atrophy in each region by a similar relative amount (e.g., 25% slowing). This has been the implicit assumption in power analyses for vMRI outcomes in neurology trials. In contrast, a non-specific effect (e.g., inflammation or fluid shift) might be expected alter the rate of atrophy in each region by a similar absolute amount. These scenarios are illustrated in Fig. 5. Examining the pattern of relative and absolute differences in volume change between treatment and control arms, plotted against the change in the control arm, over an ensemble of different brain regions may thus indicate whether the observed effects are more consistent with a disease-related or with a non-specific effect. Simple regression analysis can be used to determine the parameters illustrated in Fig. 5a, b and to indicate which scenario best explains the data. The equations describing these relationships may be generalized if, for example, there is evidence of a non-negligible y-intercept for δ_abs_ vs. % change in placebo arm.

Most neurology trials published to date have reported only 1–3 vMRI outcomes. One exception is the solanezumab EXPEDITION3 trial in Alzheimer’s disease, for which 12 vMRI outcomes were reported. The graphical analysis for EXPEDITION3 is shown in Fig. 5c, d. The regression lines indicate that in this case the overall pattern of the treatment effect on brain atrophy is most consistent with the region-proportional scenario, albeit small in magnitude, as would be expected in the case of slowing the disease trajectory.

This analysis is most straightforwardly applicable to trials with systemic administration of the therapeutic. For trials with more invasive procedures, such as intraparenchymal administration, the target region and those traversed may follow a different pattern, especially immediately following treatment. This further emphasizes the value in analyzing a number of brain regions, to be able to compare treatment effect patterns in those that are directly physically impacted by the administration process, versus those that are not.

#### Transient vs. Persistent Effects

**Fig. 6:**
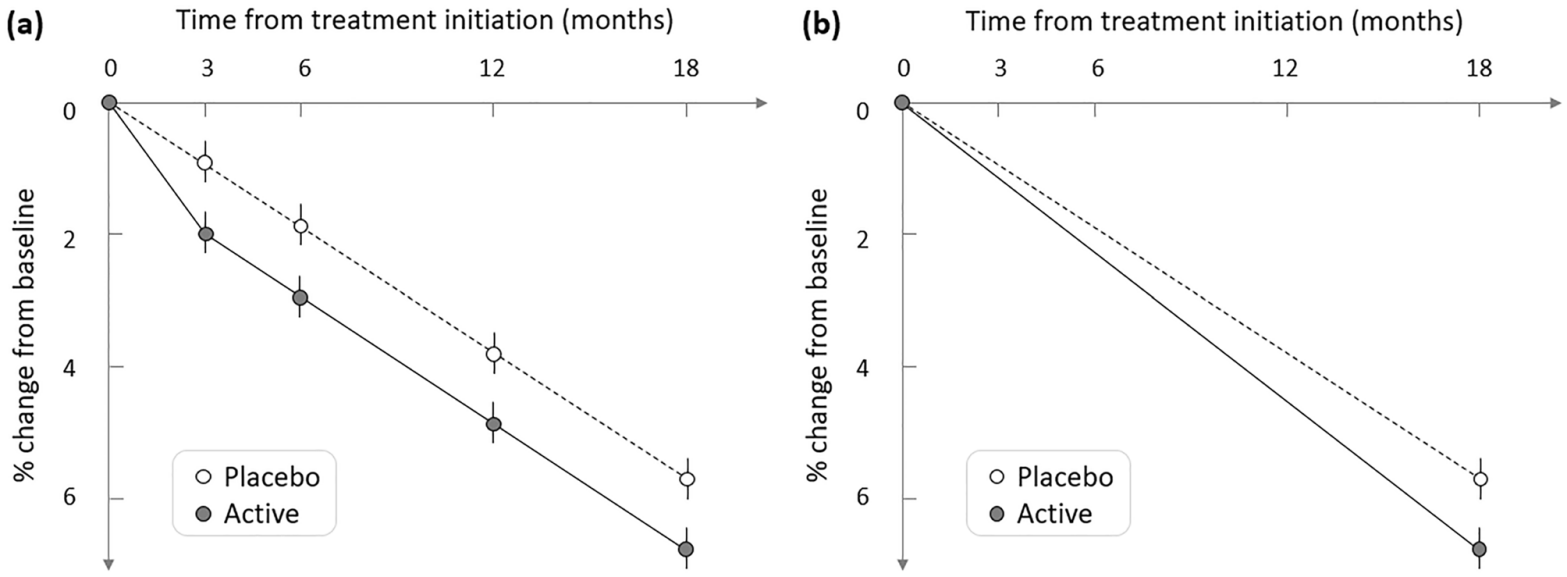
Schematic illustrating the interpretive value of intermediate scans, similar to data observed in [187]. (**a**) A transient increased volume loss relative to placebo immediately after treatment initiation, with no long-term change in the rate of atrophy, can be detected and its time course well understood if intermediate scans are acquired. (**b**) If only baseline and endpoint scans are acquired, the transient nature of the treatment effect is lost, and the data may be interpreted differently, as an ongoing acceleration of brain atrophy

Pseudo-atrophic contributions to apparent brain tissue loss elicited by treatment may exhibit a different temporal profile than “true” underlying effects on brain atrophy. For example, treatment-induced changes in inflammatory status may be transient or occur over a shorter time scale than sustained reduction in neuronal loss. One way of potentially distinguishing these contributions to apparent volumetric changes is thus to scan trial participants at intermediate time intervals during the trial and examine the temporal profile of treatment effects (Fig. 6).

Two examples from recent clinical trials illustrate the value of this approach. First, results from trials in multiple sclerosis with compounds having an anti-inflammatory action suggest that accelerated brain tissue loss is strongest in the first year after treatment initiation but becomes less apparent at later time points [181, 182]. Second, frequent vMRI scans during trials with the BACE inhibitor verubecestat in Alzheimer’s disease revealed a transient decrease in hippocampal volume loss relative to placebo immediately following treatment initiation (0–3 months) but subsequently no difference from placebo over the remaining trial period (3–18 months) [187].

#### Relationship to Anatomical Distribution of Target

If the therapeutic target is heterogeneously distributed in the brain, then local effects associated with the target pathology can potentially be identified. For example, a change in the local tissue microenvironment associated with amyloid plaques or tau tangles-associated elicited by an Alzheimer’s disease therapeutic candidate targeting one of those pathologies could result in effects on brain atrophy that reflect the underlying distribution of that pathology. Preferentially increased brain volume loss in regions with high amyloid load was recently reported for a phase 3 trial of the BACE inhibitor verubecestat in mild-to-moderate Alzheimer’s disease [187]. The increasing availability of PET tracers for other pathological targets (tau for Alzheimer’s disease, with more under development for other pathologies) will enable such effects, if present, to be revealed across different therapeutic approaches and neurological disorders.

#### Diffusion MRI: Microstructure vs. Macrostructure

Another means to help disambiguate potential confounding effects on vMRI outcomes is to acquire a diffusion-weighted MRI (DWI) sequence as part of the MRI protocol. While most of the research interest in brain DWI over the past 30 years has focused on the study of white matter (including notable white matter degeneration in several neurological disorders), more recently it has begun to be applied to the study of gray matter as well. Recent reports have started to establish a relationship between gray matter atrophy and diffusivity over the course of Alzheimer’s disease, suggesting that mean diffusivity increases with symptom severity in more familial disease [188], and tracks inversely with cortical thickness [189] and astrocytosis [189, 190]. More advanced diffusion metrics, reflecting more biologically specific aspects of cortical structure, have begun to evidence promise in multiple sclerosis and frontotemporal dementia in addition to Alzheimer’s disease [191–193]. These more advanced models may also help to protect against confounding effects of partial volume effects and contamination from surrounding cerebrospinal fluid in diffusion measures of the cortical ribbon.

In a treatment trial, deviations from relationships established in natural history data sets (and ideally confirmed in the placebo arm) may indicate possible changes in the local tissue microstructure indicative of confounding effects or inflammatory responses. At minimum, a DWI sequence sufficient to estimate basic DTI parameters (mean diffusivity, fractional anisotropy) in both white and grey matter should be appropriate for this purpose. More advanced, multi-shell DWI sequences can be acquired if the scanners used in the trial support them, as they permit more sophisticated modeling of the tissue microstructure; for example, to interrogate cortical neurite properties [191, 194] or neuroinflammation-related parameters (e.g., changes in density of glia or astrocytes) more explicitly [195].

Finally, combining structural imaging data with molecularly-specific data from synaptic [125] or neuroinflammation [76, 85] PET radiotracers is likely to be beneficial in the interpretation of observed treatment effects on brain atrophy or suspected pseudo-atrophy.

## Imaging in the Context of Fluid Biomarkers

While the scope of this article is imaging biomarkers, clinical trials are designed and conducted with a wider range of tools available, and the role of imaging is not considered in isolation. Whereas acceptable MRI scanners are widely available across many geographies, the use of PET requires radiochemistry procedures to be established local to the imaging site or the existence of a distribution network to provide tracer doses—a substantial investment. Fluid biomarkers, especially analytes obtained from cerebrospinal fluid (CSF) or blood samples, are of particular relevance to the present discussion as they often measure similar aspects of biology to imaging methods [196]. While imaging offers the ability to provide anatomical localization, fluid samples offer the advantages of enabling multiple different biomarkers to be assayed from a single procedure and generally having lower cost and more widespread availability. This is particularly true of plasma biomarkers which also offer a substantially lower participant burden than a CSF draw or imaging procedure. By way of example, CSF Aβ_1-42_ has been used as an alternative to amyloid PET in the screening phase for AD trials to accommodate geographies with differing availability of the two methods [9], and CSF markers of Aβ and tau (phosphorylated tau and total tau) have been frequently used as alternative outcome biomarkers reflecting disease pathology [5, 9, 10, 197, 198].

Recently, three major developments in the field have significantly broadened the potential scope for fluid biomarkers in the context of neurology trials. First, the gamut of well-characterized assays for different disease-relevant analytes has expanded, with a number of species reflecting protein hyperphosphorylation and/or aggregation, neuroinflammatory processes and neuronal injury becoming better understood and characterized [196, 199–207]. Assays for more specific amyloid and tau proteins are providing a more precise window on AD biology, and neurofilament light chain (NfL) appears to provide a sensitive marker of axonal injury across multiple neurological conditions. Fluid biomarkers for proteins relevant to other neurological conditions are becoming available—for example mutant huntingtin for Huntington’s disease or frataxin for Friedreich’s ataxia—although assays for some proteinopathies such as α-synuclein and TDP-43 remain a challenge. Second, the more widespread availability and site-to-site standardization of high-quality automated assays (e.g., Roche Elecsys [208]) has put the measurement of some widely-used fluid biomarkers on a firmer footing. Third, it is now possible to measure from blood plasma a number of analytes previously requiring a CSF draw; these include Aβ and tau species, along with NfL [209–215].

While much depends on the specific performance of the various imaging and fluid assays, and their context of use in clinical trials, the strengths and weaknesses of each modality can shape their role. For example, a plasma marker that does not perform quite as well as an imaging marker may still have value at an earlier point in the screening cascade to cheaply exclude individuals that are likely to screen negative on a subsequent PET scan. In this way, the PET scan would be confirmatory with a low likelihood of expensive screening failures, and if PET is being tracked longitudinally then the PET scans would have additional value as baseline measurements. Looking further ahead to when plasma biomarkers perform as well as PET scans for both screening and detecting treatment effects, the role of imaging markers might be more prioritized towards questions relevant to anatomical distribution, and use in smaller, early-phase trials.

Nevertheless, it must always be remembered that any biomarker measures what it measures—analytes detected in blood plasma may have some relationship to the substrates of PET ligand binding sites in the brain, or to imaging measures of neurodegeneration, but they are not the same thing. For example, a fluid analyte may more closely reflect the current circulating concentration of a given molecule (in some compartmental relationship with brain parenchyma) whereas a “corresponding” PET scan may reflect the accumulated levels of related aggregates. For certain purposes, such as AT(N) staging in AD, one may substitute for the other at the conceptual level of that framework, but the performance may differ [216, 217]. With respect to the detection of treatment effects, at the present time it remains valuable to obtain convergent evidence of treatment effects on different markers of molecular pathology and neurodegeneration.

## Conclusions

Imaging biomarkers play a wide-ranging role in clinical trials for neurological disorders. This includes selecting the appropriate trial participants, establishing target engagement and mechanism-related pharmacodynamic effect, monitoring safety, and providing evidence of disease modification. In the early stages of clinical drug development, evidence of target engagement and/or downstream pharmacodynamic effect—especially with a clear relationship to dose—can provide confidence that the therapeutic candidate should be advanced to larger and more expensive trials, and can inform the selection of the dose(s) to be further tested, i.e., to “de-risk” the drug development program. In these later-phase trials, evidence that the therapeutic candidate is altering disease-related biomarkers can provide important evidence that the clinical benefit of the compound (if observed) is grounded in meaningful biological changes. The interpretation of disease-related imaging markers, and comparability across different trials and imaging tools, is greatly improved when standardized outcome measures are defined. This standardization should not impinge on scientific advances in the imaging tools per se, but provides a common language in which the results generated by these tools are expressed. PET markers of pathological protein aggregates and structural imaging of brain atrophy are common disease-related elements across many neurological disorders. However, PET tracers for pathologies beyond amyloid β and tau are needed, and the interpretability of structural imaging can be enhanced by some simple considerations to guard against the possible confound of pseudo-atrophy. Learnings from much-studied conditions such as Alzheimer’s disease and multiple sclerosis will be beneficial as the field embraces rarer diseases.

## Supplementary Information

Below is the link to the electronic supplementary material.

Supplementary file1 (PDF 1225 KB)
